# Evaluating emergency preparedness and impact of a hurricane sandy in pediatric patients with diabetes

**DOI:** 10.1186/s40696-016-0012-9

**Published:** 2016-02-03

**Authors:** Rubina Heptulla, Rebecca Hashim, Doreen Newell Johnson, Jeniece Trast Ilkowitz, Gina DiNapoli, Venkat Renukuntla, Jennifer Sivitz

**Affiliations:** 1 Pediatric Endocrinology and Diabetes, Children’s Hospital at Montefiore and Albert Einstein College of Medicine, 3411 Wayne Ave, Suite: 4 M, Bronx, NY 10467 USA; 2 Department of Psychiatry and Pediatrics, Children’s Hospital at Montefiore, Bronx, NY USA; 3 Pediatric Endocrinology and Diabetes, Children’s Hospital at Montefiore, Bronx, NY USA; 4 Pediatric Endocrinology and Diabetes, Albert Einstein College of Medicine, Bronx, NY USA; 5 Pediatric Endocrinology and Diabetes, Hackensack University Medical Center, Hackensack, NJ USA

**Keywords:** Disaster, Preparedness, Hurricane, Diabetes, Pediatrics

## Abstract

**Background:**

Natural disasters have always been associated with significant adverse events including medical and mental health problems. Children with chronic disease such has diabetes have also been believed to be affected to a greater extent by any natural disaster. The purpose of this study was to assess and compare emergency preparedness post-disaster and post-traumatic stress effects of Hurricane Sandy in affected and relatively unaffected populations.

**Methods:**

The study was conducted between February and July 2013. A total of 142 families caring for children with Type 1 Diabetes Mellitus (T1DM) who attended clinics were recruited from hospitals in Bronx, NY (control) and in NJ (affected) by Hurricane Sandy. Subjects were recruited to participate in a survey 3–6 months after the hurricane. Data on demographics, glycemic control and insulin regimens were collected. Families were surveyed for socio-economic status (SES), using Hollingshead questionnaire, general and diabetes preparedness and the Hurricane Related Traumatic Experiences (HURTE) questionnaire was used to evaluate for symptoms of post-traumatic stress.

**Results:**

Ninety-five percent of families reported to be generally well to moderately prepared for the hurricane and 83 % reported to be very well prepared with regards to their child’s diabetes during the disaster. There was no difference between the sites for preparedness for the disaster, age or gender. There was a trend toward significance (p < 0.06) in New Jersey subjects as to a greater psychological impact from the hurricane. Poor glycemic control was significantly associated with lower SES (p < 0.008). Most importantly, SES was unrelated to preparedness for diabetes management during the hurricane.

**Conclusions:**

Despite low SES, families were generally well to moderately prepared for hurricane. In children with diabetes, interventional studies should be designed and implemented so that glycemic control remains unaffected, following any major disaster.

## Background

The International Federation of Red Cross and Red Crescent Societies categorize a disaster as an event that causes more than ten deaths, affects more than 100 people or leads to an appeal by those affected for assistance [1]. Disasters can further be classified as natural or manmade. Naturally occurring disasters include hurricanes, tornadoes, floods and earthquakes, whereas fires, mass transportation incidents, events involving environmental toxins and episodes of civil unrest are considered manmade disasters [2]. The Federal Emergency Management Agency (FEMA) recommends that families should be self-sufficient for greater than or equal to 3 days after a disaster occurs [3]. Disaster preparedness reduces fear, anxiety and losses that may occur as a result of disasters. The importance of disaster preparedness is highlighted by recent weather events, such as Hurricane Katrina and the terrorist attacks of September 11, 2001. According to FEMA, the importance of disaster preparedness is only becoming more relevant as the frequency of natural disasters has increased over the past 50 years. On average, over the last 10 years there have been 65.1 declared disasters per year (2004–2013) compared to 14.7 declared disasters per year between 1954–1963 [3].

Management of chronic diseases, such as diabetes, is challenging during and after disasters. Furthermore, the psychological impact of a disaster may have negative consequences for patients as seen after Hurricane Katrina [4]. Diabetes affects approximately 3.4 million children in the United States [5]. Recent data suggests approximately one out of 500 children in the US have type 1 diabetes (T1DM) by 18 years of age [5]. Given the vulnerability of children to environmental factors (toxins/extreme temperatures) and the multiple resources needed to care for someone with T1DM (i.e. access to adequate food and water, insulin, testing supplies such as meters, glucose test strips, and lancets, etc.), disaster preparedness becomes absolutely essential. The Board of Directors of the American Academy of Pediatrics further emphasized this need when they identified disaster preparedness as one of seven priority issues requiring special attention and resources [2].

In recent years, there have been a number of disastrous events that have tested our readiness for such situations. Currently, we are reeling from the aftermath of Hurricane Sandy, the largest Atlantic Hurricane on record, which made landfall on October 29, 2012. The hurricane, also known as Super Storm Sandy, caused widespread destruction in the areas of New York City, Long Island, Westchester County of New York State, New Jersey, and Connecticut. Services for health care delivery, hospitals and pharmacies were significantly impacted during this time [6, 7]. The influence of disaster has effects that are more chronic and can have a negative impact on children. Furthermore, inadequate preparation prior to the storm can affect both the immediate and long-term consequences of diseases such as diabetes [8, 9]. Our previous research suggested that patients with T1DM who were surveyed before the disaster were inadequately prepared for a disaster [10]. In this trial, we examined the after-effects of a disaster in families that were directly affected and those that were unaffected and lived in the same geographic area hit by Hurricane Sandy.

## Methods

The study was approved by the Institutional Review Board (IRB) at both participating sites. The protocol was expedited and the participants were consented before administering the surveys. After Hurricane Sandy in New York and New Jersey, a survey was designed to determine the level of disaster preparedness and was distributed to families caring for children with T1DM who were attending the diabetes clinic at an urban hospital in Bronx, New York, which was minimally affected by Hurricane Sandy; and the diabetes clinic at a suburban hospital in Hackensack, New Jersey, which was severely affected by the hurricane. Severely affected areas were characterized by loss of services such as electricity, water and phone as well as destruction of homes, businesses and schools for many days, weeks or months. This includes damage caused by flooding, fires, and high winds. Severely affected areas required people to leave their homes before or after the storm to remain safe [11, 12]. Minimally affected areas had less destruction, for example trees falling, and had loss of services (such as electricity and transportation) for only a few hours or days [13]. Due to the media coverage, people were very aware of what areas were more and less affected. The survey was administered during routine clinic visits to English-speaking families. Adults 18 years of age and older did not need to be accompanied by a legal guardian to answer the questions in the survey and those under 18 filled out the survey with their legal guardian. The sample (subjects) was a convenience sample of families attending one of the two clinics described. Each family was approached individually in the clinic and was asked to complete the survey using paper and a pencil. On average, families took approximately 15–20 min to complete the survey. We collected a total of 142 surveys: 77 from hospital in the Bronx and 65 from the hospital in New Jersey. We approached 150 families and 142 agreed to participate. The eight families who choose not to participate did not specify the reason.

The survey included four parts: (1) preparation for a disaster in general, (2) preparation for caring for the child with diabetes in the event of a disaster, (3) demographic characteristics and (4) a measure to assess hurricane-related traumatic experiences. The questions in the first and second parts were graded on a scale of one to five points as described in our previous work [10], weighted according to their degree of importance in disaster preparedness. The general and diabetes preparedness raw scores were divided into tertiles, to indicate well, moderate and poor preparedness. The first set of questions on the survey elicited an estimation of level of general preparedness. A disaster supply kit, or a 72-hour emergency kit, consisted of 3-days supply of food, water and current prescriptions, and a first aid kit which includes non-prescription treatments for common illnesses and tools. The disaster supply kit and its different elements accounted for 44 of the 70 possible points awarded for the general preparedness portion of the survey. Further points were awarded for additional components of a family disaster plan such as identified emergency contact numbers, household smoke alarms and fire extinguishers, in addition to communication and evacuation plans. A score of 0–23 reflected poor preparedness, 24–47 reflected moderate preparedness and 48–70 reflected well preparedness for an emergency or disastrous situation.

For the next part of the survey, questions were designed to evaluate diabetes preparedness in an emergency or disaster as previously described [10]. The survey addressed items and skills necessary to care for a child with diabetes including: wearing a medical alert bracelet, completion of diabetes education classes, maintenance of current blood glucose logs, possession of a glucagon emergency kit, at least 3 days supply of current prescriptions (insulin, insulin syringes/insulin pens and pen needles, glucose meter, glucose test strips, lancets and lancing device and insulin pump supplies if using an insulin pump) and up to date immunizations. One could earn up to 40 points on this portion of the survey. A total of zero to 13 points was considered poorly prepared, 14–27 indicated moderate preparation, and 28–40 points was considered well prepared to manage a child with diabetes in the setting of an emergency or disaster.

Demographic characteristics were elicited in the third section of the questionnaire. Data was collected on gender, race and the child’s age. Hemoglobin A1C (HbA1C) level at the visit as well as the child’s current insulin regimen was also obtained. HbA1C was further defined as well controlled if HbA1C was <7.5 %, moderately controlled if 7.6–8.9 % and poorly controlled if >9 %. The Hollingshead 4-Factor Index of Social Status was used to determine the estimated socioeconomic status (SES) of the families [14]. A calculation based on education and occupation levels was used to determine SES status. Education and occupation levels were self-reported by the families interviewed. A total score range of 8–66 could be earned in this section. A score of 48–66 was considered high SES, 28–47 was moderate and 8–27 was considered low SES.

Finally, Hurricane Related Traumatic Experiences (HURTE) was used to measure hurricane-related traumatic experiences as previously described [15]. In brief, HURTE elicited the children’s reports of experiencing life threatening events and perceived loss and disruption [16]. The items on the HURTE were developed to identify potentially confirmed events and answered with a “yes” or “no.” There was one question that addressed whether or not the child felt they were in a life threatening position during the event. The next six items inquired about witnessed events that were reflective of life threatening events and the sum of these questions was used to determine a score of *n* for life threatening events during a hurricane. Finally the last ten items regarded loss and disruption. These items were also totaled to obtain a score of *n* for loss and disruption. Each “yes” response counted as one point and each “no” response counted as zero points. (See Appendix 1).

Statistics: The advanced model of SPSS 16.0 was used to perform statistical analyses (SPSS, Chicago, IL). Figures and graphs were obtained using both SPSS 16.0 and Graphpad Prism 6.0 (GraphPad, La Jolla, CA). Unless otherwise indicated, all data is expressed as means and SDs or proportions. The analyses included: cross-tabulation independent-sample *t* tests, Mann–Whitney test and analysis of variance and Pearson correlation. Significance was considered at *p* < 0.05 level.

## Results

One hundred and forty-two subjects were surveyed from two centers (N = 77 from the Bronx, New York and N = 65 from Hackensack, New Jersey). The patient ages were well matched at the two sites (Table 1), 74 were females and 68 were males, mean HbA1c was 8.8 ± 1.9 % (24 % were well controlled, 40 % were moderately controlled and 36 % were poorly controlled). Subjects were primarily managed on insulin pumps (56 %) or multiple daily insulin injections with long acting and rapid acting analog at meals (40 %). The remaining was on two injections per day, using a combination of intermediate and rapid acting insulin analogs. Socioeconomic status (SES) of the cohort showed that 28 % were in the lower economic strata and 31 and 38 % were in the moderate to high economic strata, respectively. Two subjects did not divulge information on their SES. SES was significantly higher at the New Jersey site and the subjects at that site had better glycemic control (Table 1). Table 1 also shows marked differences in race at the two sites. We divided the race based on different socioeconomic status. For subjects between Bronx (New York) and New Jersey, we did see a significant difference between the race and the socioeconomic status. In New Jersey, there were significant number of subjects that belonged to the high and moderate socioeconomic status, when compared to the population in Bronx, New York (NJ vs. Bronx, p = 0.001). Compared to New Jersey, the diabetes preparedness was slightly better in subjects with low socioeconomic status in Bronx, New York (p = 0.03). There were no other differences that were noted regarding general preparedness (p = 0.61, NS) or diabetes preparedness (p = 0.76, NS) at either site despite socio-economic disparities. Further, there were no statistically significant differences between the HURTE scores (for post-traumatic stress events) and the different socioeconomic status at either site (p = 0.36).

**Table 1:** Patient characteristics at the two participating sites (Hackensack, NJ, and Bronx, NY)—patient characteristics include age in years, HbA1C  %, race, gender, Hollingshead (SES status), past history of facing disaster, general preparedness and diabetes preparedness

	Hackensack, NJ (n = 65)	Bronx, NY (n = 77)	Total	Significance (p value)
Age (years)				
Mean ± SD	13.3 ± 2	13.3 ± 2	13.3 ± 2	NS
HbA1C %				
Mean ± SD	8.2 ± 1	9.3 ± 2.3	8.8 ± 1.9 % well controlled 24 % moderately controlled 40 % poorly controlled 36 %	<0.008
Frequencies (%) race				
Caucasian	75 (n = 49)	12 (n = 9)	41 (n = 58)	
African American	5 (n = 3)	22 (n = 17)	14 (n = 20)	<0.0001
Hispanic	14 (n = 9)	58 (n = 45)	38 (n = 54)	
Mixed	6 (n = 4)	8 (n = 6)	7 (n = 10)	
Gender				
Female	38 (49 %)	36 (55 %)	74 (52 %)	
Hollingshead (SES status)	45 ± 14	33 ± 16	39 ± 15	<0.0001
SES (frequency %)				
High	53 (n = 35)	27 (n = 21)	39 (n = 56)	<0.002
Moderate	36 (n = 23)	29 (n = 22)	31 (n = 45)	<0.001
Low	11 (n = 7)	44 (n = 34)	29 (n = 41)	0.31
Past history of facing disaster (frequency %)				
No history	1 (n = 1)	0	7 (n = 1)	
History of one	63 (n = 41)	66 (n = 51)	65 (n = 92)	NS
More than one	36 (n = 23)	34 (n = 26)	35 (n = 49)	
General preparedness (frequency %)				
High	39 (n = 25)	52 (n = 40)	46 (n = 65)	
Moderate	61 (n = 40)	42 (n = 32)	51.5 (n = 72)	NS
Low	0	6 (n = 5)	4 (n = 5)	
Diabetes preparedness (frequency %)				
High	85 (n = 55)	82 (n = 63)	83 (n = 118)	NS
Moderate	15 (n = 10)	14 (n = 11)	15 (n = 21)	NS
Low	0	4 (n = 3)	2 (n = 3)	0.03

**Fig. 1:**
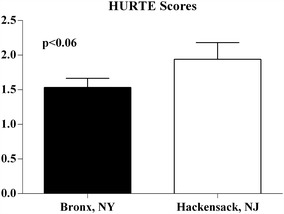
HURTE (Hurricane Related Traumatic Experiences) Scores—Hurricane Related Traumatic Experiences (HURTE) was used to measure hurricane-related traumatic experiences which elicit the children’s reports of experiencing life threatening events and perceived loss and disruption. The questions on the HURTE were developed to identify potentially confirmed events and answered with a “yes” or “no”

In Fig. 1, the HURTE scores showed that the subjects in New Jersey had more symptoms of post-traumatic stress than the Bronx patients. There was a trend toward significance (p < 0.06).

## Discussion

In this cross-sectional study, we compared two populations in the area where a major Hurricane occurred on October 29, 2012. Hackensack, NJ was more severely affected than the Bronx, NY. We showed that despite differences in SES and glycemic control in the two patient populations, general and diabetes emergency preparedness was similar for the two populations except for the diabetes preparedness in low SES population in Bronx, NY which was slightly better than the low SES population in Hackensack, NJ. There was a trend towards post-traumatic stress symptoms in the New Jersey population after the storm, perhaps because they were more affected by the disaster than the Bronx.

These study results were surprising because, of a previous study done in Texas, where we have shown that poor glycemic control is associated with lower general and diabetes preparedness [10]. Those finding may have been due to storm lethargy, the idea that many storms are predicted in general and that people are asked to prepare for it. However, even in low SES populations particularly New York, emergency preparedness, especially for diabetes, was high. This is very reassuring that patients with diabetes are taking heed to warnings and have adequate supplies and report that they were prepared. The reason for storm preparedness may be due to community support, or the urban setting versus the rural setting in Texas. Emergency preparedness in the low SES population should be explored more. However, in concordance with our previous findings, general preparedness scores were lower than diabetes preparedness. For diabetes patients it is not only important to have diabetes supplies but adequate supplies of food and water as well.

In a year with multiple disasters, our past research on disaster preparedness suggested hurricane fatigue and lower preparedness over time. In 2012, the only major storm to hit the Northeastern Unites States was Hurricane Sandy and perhaps it is possible that the low frequency of such incidents was the reason for good emergency preparedness, as compared to our survey in Texas when there were many powerful storms that were predicted but did not happen.

SES had no impact on diabetes preparedness and that is also contrary to our previous finding. SES continues to have an impact on overall glycemic control as previously reported within the US and outside [17–19]. Exploring why patients with low SES were prepared for storms may give insight into how to improve glycemic control in this population as well.

In this study, we also wanted to study the post-traumatic effects of the disaster and hence we compared two populations. New Jersey was more severely affected than the Bronx, and thus in looking at these two populations we could evaluate the psychological effects of the disaster. The study suggested that there was a trend toward more psychological symptoms in the New Jersey population compared to the Bronx. This is consistent with other disasters, such as the earthquakes in Japan [20, 21] and can affect both glycemic control and blood pressure management [22]. Additionally, the HURTE questionnaire could have been affected by the perceived fear of potential damage in the less affected group due to the exposure to media coverage of the disaster in the neighboring area.

The limitations of this study were that only two locations were surveyed and the most severely affected areas were not surveyed. It is possible that if we had surveyed the more affected areas, we would have noted a more profound effect on post-traumatic stress related events. However, we note that even in moderately affected areas there is a psychological impact of disasters. Since this is a cross-sectional study it is not possible to have a cause and effect of disasters. There is also a recall bias associated with this kind of study since it is post-impact. In future, we will do a baseline evaluation of our patient population and compare it to an evaluation done after a disaster has occurred. Despite the limitations of the study, we have uncovered important factors that we previously thought affected disaster preparedness, like poor glycemic control and SES, and found them to not have an effect in this study.

## Conclusions

In conclusion, preparing for a disaster is important for diabetes care and interventional studies to address the stress following disasters should be designed and ready to be implemented so that glycemic control remains unaffected and patients that are impacted by a major disaster are able to cope with the aftermath.

## Abbreviations

FEMA: Federal Emergency Management Agency
SES: Socio-economic Status
T1DM: Type 1 Diabetes Mellitus
HURTE: Hurricane Related Traumatic Experiences
